# CD2 Regulates Pathogenesis of Asthma Induced by House Dust Mice Extract

**DOI:** 10.3389/fimmu.2020.00881

**Published:** 2020-05-12

**Authors:** Tanwir Hashem, Ananth K. Kammala, Kanedra Thaxton, Ryan M. Griffin, Kellie Mullany, Reynold A. Panettieri, Hariharan Subramanian, Rupali Das

**Affiliations:** ^1^Department of Physiology, College of Natural Science, Michigan State University, East Lansing, MI, United States; ^2^College of Human Medicine, Michigan State University, East Lansing, MI, United States; ^3^Department of Chemical Engineering and Material Science, College of Engineering, Michigan State University, East Lansing, MI, United States; ^4^Rutgers Institute for Translational Medicine and Science, New Brunswick, NJ, United States

**Keywords:** asthma, Th2-allergic response, house dust mite extract, costimulatory molecules, CD2, interleukin (IL)-13, microRNAs

## Abstract

Characteristic of allergic asthma, CD4+Th2 lymphocytes secrete Th2 cytokines, interleukin (IL)-4, IL-13, and IL-5 that mediate the inflammatory immune response. Surface expression of CD2 and its ligand, CD58, is increased on the monocytes and eosinophils of asthma patients, which correlate with elevated serum IgE levels, suggesting that CD2 may contribute to allergic airway inflammation. Using a murine model of asthma, we observed that house dust mice extract (HDME)-exposed Balb/c mice have increased airway hyperresponsiveness (AHR), lung inflammation, goblet cell hyperplasia, and elevated levels of Th2 cytokines in the lungs, as well as increased serum IgE levels as compared to the control mice. In contrast, with the exception of serum IgE levels, all the other parameters were significantly reduced in HDME-treated *Cd2*^−/−^ mice. Interestingly, *Il13* but not *Il4* or *Il5* gene expression in the lungs was dramatically decreased in HDME-exposed *Cd2*^−/−^ mice. Of note, the gene expression of IL-13 downstream targets (Muc5b and Muc5ac) and high affinity IL-13Rα2 were upregulated in the lungs of HDME-exposed Balb/c mice but were significantly reduced in HDME-exposed *Cd2*^−/−^ mice. Consistently, gene expression of microRNAs regulating mucin production, inflammation, airway smooth muscle cell proliferation and IL-13 transcripts were increased in the lungs of HDME-exposed *Cd2*^−/−^ mice. Given the established role of IL-13 in promoting goblet cell hyperplasia, lung inflammation and AHR in allergic asthma, our studies reveal a unique role for CD2 in the regulation of Th2-associated allergic asthma.

## Introduction

Asthma, a chronic inflammatory disorder, can manifest with high serum IgE levels, AHR, lung inflammation, goblet cell hyperplasia and airway remodeling (1, 2). Allergic asthma is often triggered by sensitization of the airways to environmental allergens such as pollens, house dust mite, pet dander, cockroaches and mold (2). In Th2-high allergic asthma, CD4+ T helper cells (Th0) differentiate into Th2 cells and make copious amounts of cytokines including interleukin (IL)-4, IL-5, and IL-13 (3) that further drive the pathogenesis of this disease. Optimal T cell activation requires signals delivered via the TCR and several co-stimulatory molecules. The best characterized co-stimulatory molecule CD28, binds to its ligands CD80 or CD86 which is critical for initial T cell activation and differentiation (4). Upon activation, several other co-stimulatory molecules are expressed on T cells that interact with their respective ligands on antigen presenting cells to sustain, enhance or inhibit the immune response in asthma (4).

CD2 is a part of the immunoglobulin (Ig) superfamily of receptors that is constitutively expressed on T cells and binds with high affinity to its ligand CD58 in humans, and CD48 in mice (5). Although earlier studies suggested that CD2 and CD28 have redundant functions in T cell activation (6), recent reports demonstrate that CD2 triggers a potent proximal T cell signaling response, whereas CD28 co-stimulation leads to activation of downstream transcription factors (7). Moreover, CD2 and CD28 co-stimulation produce distinct functional responses (7). CD58 is highly expressed on the eosinophils of asthmatic patients (8) and increased expression of FcεRI on CD2^high^ monocytes from asthma patients correlates with higher serum IgE levels (9). While these studies provide a causal link between CD2 and asthma, the specific role of CD2 in allergic asthma remains to be defined.

In the current study, we investigated the role of CD2 in a murine model of allergic asthma using mice deficient in CD2 (*Cd2*^−/−^). Exposure of WT Balb/c mice to an airborne allergen such as HDME induced a quintessential allergen-induced AHR, inflammation and goblet cell hyperplasia in association with high levels of serum IgE, Th2 cytokines and mucus production. While the cardinal features of allergen-induced inflammation was abrogated in *Cd2*^−/−^ mice, our studies reveal that CD2 specifically regulates IL-13 production but does not affect other hallmark Th2 cytokines, including IL-4, IL-5, and IL-10. Accordingly, our studies reveal that CD2 promotes allergen-induced mucus production but not IgE levels. Mechanistically, we demonstrate that CD2 critically modulates gene expression of alarmins such as IL-25, IL-13 receptors (IL-13Rα1 and IL-13Rα2) and several microRNAs that have known roles in the pathogenesis of allergic asthma.

## Materials and Methods

### Mice

Balb/c mice were purchased from Jackson Laboratories (Bar Harbor, ME). *Cd2*^−/−^ mice on a C57BL/6 (B6) background was a kind gift from Dr. Dorothy Yuan (University of Texas Southwestern Medical Center, Texas). *Cd2*^−/−^ mice on a Balb/c background were obtained by backcrossing B6. *Cd2*^−/−^ mice with Balb/c mice for 9 generations. All animal studies were approved by the Institutional Animal Care and Use Committee at the Michigan State University.

### Mouse Model of Asthma

Balb/c (wild-type) and *Cd2*^−/−^ age-matched (10–11 weeks old) female mice were injected intranasally (i.n) with 25 μl of house dust mite extract (HDME) (Greer Laboratories, Lenoir, NC) or 25 μl of PBS on alternate days for a total of 7 injections. HDME was reconstituted in sterile PBS at a concentration of 2 μg of protein/μl. The amount of HDME protein, Derp1 and endotoxin was 50 μg, 3.1 μg and 38.4 EU, respectively per injection in a volume of 25 μl. Twenty-four hours after the final HDME challenge, mice were anesthetized for measurement of airway hyperresponsiveness (AHR) and then sacrificed for collection of blood, bronchoalveolar lavage fluid (BALF) and lung tissue for various endpoint analysis.

### AHR Assessment

Mice were anesthetized with a cocktail of 100 mg/kg ketamine (Henry Schein Animal Health, Dublin, OH), 10 mg/kg xylazine (Akorn, Lake Forest, IL) and 3 mg/kg acepromazine (Henry Schein Animal Health, Dublin, OH) through intraperitoneal (i.p.) injection and tracheostomized. Airway mechanics was measured using forced oscillation technique by flexiVent, a small animal ventilator (SCIREQ®, Quebec, Canada). Parameters of AHR such as airway resistance (Rrs) and elastance (Ers) were assessed by a methacholine (MCh; Sigma-Aldrich, St. Louis, MO) challenge test with increasing doses of MCh as indicated in Figure 2.

### Blood Serum Collection

Blood was collected from the superior mesenteric vein of the mouse and left at 4°C overnight. Serum was collected the next day and analyzed for total IgE and HDME-specific IgE using commercially available ELISA kits from Invitrogen (Carlsbad, CA) and Chondrex (Redmond, WA), respectively.

### Evaluation of Lung Inflammation and Goblet Cell Hyperplasia

The lungs were infused via the trachea with 10% buffered formalin. After excision, the lungs were immersed in fresh 10% formalin overnight. Samples were then embedded in paraffin, cut into 5-μm-thick sections and stained with hematoxylin and eosin (H&E) or Periodic acid-schiff (PAS). Digital images of sections were obtained using a Nikon Eclipse 50i microscope (Nikon, Japan) equipped with a INFINITY-3 digital color camera (Lumenera Corporation, Canada), and INFINITY ANALYZE 6.5.4 software.

### PAS Scoring

Goblet cell hyperplasia was evaluated on PAS–stained lung sections. Each lung sample was divided into 9 imaginary sections and digitally imaged at 10X magnification in an effort to consistently observe identical regions across all samples and experiments. The intensity of PAS staining was assessed using ImageJ software (NIH, Bethesda, MD) to determine PAS positive cells as well as the percent area of PAS positive cells in each section.

### Quantitative Real-Time PCR

Lungs were dissociated in TRIzol solution (Thermo Fisher Scientific, Waltham, MA) using a high-speed homogenizer (Fisher Scientific, Hampton, NH) and total RNA was extracted as per manufacturer's protocol. RNA (2 μg) was reverse transcribed into cDNA using SuperScript III in a 20 μl reaction volume or using the Taqman Advanced miRNA cDNA synthesis kit (for microRNA analysis) according to the manufacturer's instructions (Applied Biosystems, Foster City, CA). Real-time quantitative PCR was performed using Quant Studio™ 3 system (Applied Biosystems) with validated Taqman primers and Fast Advanced Master Mix according to manufacturer's instructions. Relative gene expression data (fold change) between samples was accomplished using the 2^−ΔΔCt^ method. GAPDH (for gene expression) or 18S (for miRNA analysis) was used as the internal reference control.

### Isolation of Immune Cell Populations From the Lungs

Lung samples were digested with collagenase P (1 mg/ml, Roche Diagnostics, Indianapolis, IN) at 37°C for 30 min. Single cell suspension was obtained by passing the digested tissue through a 70 μm cell strainer (Alkali Scientific Inc, Fort Lauderdale, FL) with a plunger. Lung mononuclear cells were then isolated using density centrifugation with Percoll (GE, Piscataway, NJ). The isolated cells were washed and resuspended in RPMI media (Life Technologies, Carlsbad, CA) conditioned with 10% FBS (Atlanta Biologicals, Flowery Branch, GA) and 1% penicillin-streptomycin (Mediatech Inc, Manassas,VA).

### Antibodies and Flow Cytometry

The antibodies used for immunofluorescence staining include CD4, CD11b, CD80, B220, DX5, TCR-β, and SiglecF (BD Biosciences). CD2, CD8, CD11c, CD28, CD40, CD48, CD86, CTLA-4, ICOS, IA/IE (MHCII), Ly6G, OX-40, and 4-1BB were from Biolegend, San Diego, CA. The catalog number and clone for each of the antibody used is included in Supplemental Table 2. Fluorochrome conjugated CD1d-tetramer (CD1d-Tet) loaded with glycolipid antigen (PBS57), or unloaded controls were provided by the NIH Tetramer Core Facility (Emory University, Atlanta, GA). Data was collected on a LSRII flow cytometer (BD Biosciences) and analyzed using FlowJo software (FlowJo LLC, Ashland, OR).

### Total and Differential Leukocyte Count From Bronchoalveolar Lavage Fluid (BALF)

Mice were euthanized and whole lung was lavaged with 0.6 ml of sterile PBS three times and the resultant BALF was centrifuged to separate the cellular components from the supernatants. Cell-free BALF was analyzed for cytokines including, IL-4 (BD Biosciences, San Diego, CA) and IL-13 (Invitrogen) by ELISA. Total BALF cellularity was determined using a hemacytometer, and the BALF composition was evaluated morphologically following differential staining. Briefly, 50,000 cells were cytospun onto a clean glass slide, air- dried and then stained with Giemsa Wright stain (Sigma-Aldrich, St. Louis, MO) for 3 min. The stained slides were washed with distilled water two times and dried. The slides were then dipped in xylene (Avantor, Radnor Township, PA) for 2 s and a cover slip was placed immediately over the cells. For each sample, a total of 200 cells were counted at 40X magnification and the number of monocytes, eosinophils, neutrophils, and lymphocytes was enumerated.

### Human Lung Samples

Lung samples from control subjects who died from other causes and individuals who died from complications of asthma were obtained from either the International Institute for the Advancement of Medicine (IIAM) or National Disease Resource Interchange (NDRI) and their use was approved by Institutional Review Board at Rutgers University. All donor tissue samples were harvested anonymously and de-identified. All patients died from asthma and as such had very severe disease. The donors were age- and gendered matched. The available information is provided in Supplemental Table 1.

### Statistics

Statistical significance was determined using GraphPad Prism software (GraphPad, San Diego, CA). Student's *t*-test with Welch's correction or two-way ANOVA was used as indicated in the figure legends. Significance is shown as ^*^(*p* < 0.05), ^**^(*p* < 0.01), or ns for non-significant values.

## Results

### CD2 Is Expressed in the Lung Tissues of Human Asthmatic Patients and Mice Exposed to HDME

**Figure 1:**
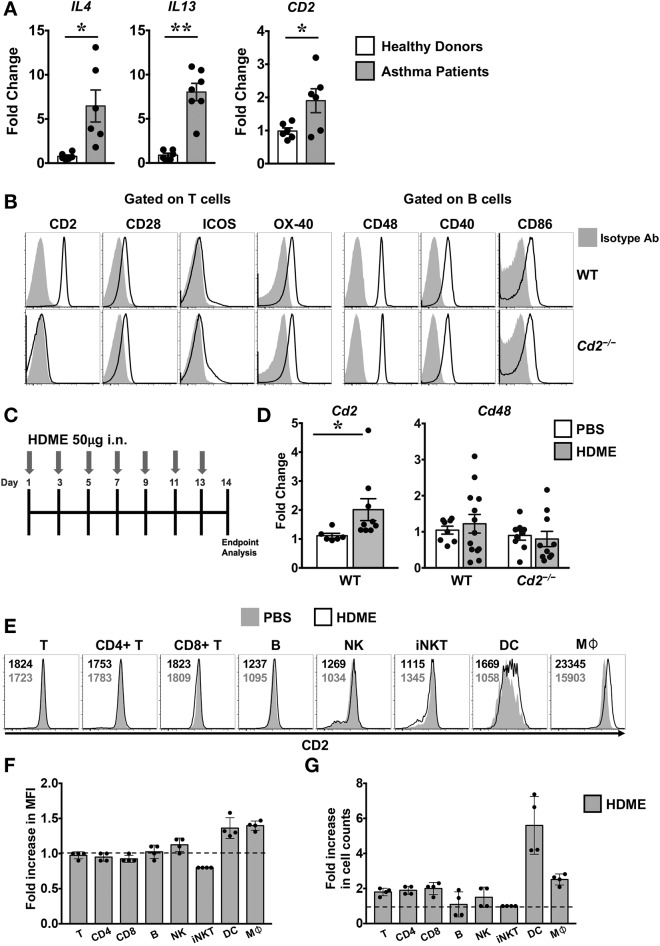
Increased expression of CD2 in the lungs of human asthmatics and HDME-challeged mice. **(A)** Whole lung tissue from healthy donors and asthma patients were analyzed for gene expression of *IL-4, IL-13* and *CD2*. Data is shown as mean fold change ± SEM with a total of 6–7 samples per cohort. **(B)** Surface expression of various co-stimulatory molecules (CD2, CD28, ICOS, and OX-40) gated on T cells and ligands for costimulatory molecules (CD48, CD40, and CD86) expressed on B in the lungs of naïve Balb/c (WT) and Balb/c. *Cd2*^−/−^ (*Cd2*^−/−^) mice. Data are representative of at least 4 mice per genotype. **(C)** Schematics of the asthma model used in the study. Mice were challenged intranasally (i.n.) with HDME on alternate days for a total of 7 injections. **(D)** Lung tissues of WT and *Cd2*^−/−^ mice injected with PBS or HMDE were analyzed for *Cd2* (left) and *Cd48* (right), as indicated in the figure. Data is presented as mean fold change ± SEM and is pooled from 3 independent experiments with a total of 8–13 mice per cohort. Statistical significance in **(A,E)** was determined by using Student's unpaired *t*-test with Welch's correction. *p ≤ 0.05, **p ≤ 0.01. **(E–G)** Mice were challenged intranasally (i.n.) with HDME for a total of 3 injections. Two hours after the last injection, mice were sacrificed and lung tissues were analyzed for surface expression of CD2 by flow cytometry. Representative histograms depict CD2 expression on various immune cells (as indicated in the figure, **E**). Numbers in the histograms indicate mean fluorescence intensity (MFI) of CD2 in HDME-treated (black font, top) and PBS-injected (gray font, bottom) mice. Fold increase in the MFI of CD2 **(F)** and cell counts **(G)** of each immune cell type in the lungs of HDME-treated mice as compared to PBS controls are shown. Dotted lines in **(E,F)** represent the baseline set at 1 for the control group. Data is presented as mean fold change ± SEM and is pooled from 4 mice per cohort.

Th2 high asthma endotye is associated with elevated levels of Th2 cytokines such as IL-4 and IL-13 (2). Accordingly, we observed that the gene expression of both *IL4* and *IL13* were significantly upregulated in the lungs of human asthma patients as compared to those from healthy donors (Figure 1A). *CD2* gene transcripts were also significantly elevated in the lungs of these asthma patients (Figure 1A), consistent with prior reports demonstrating that CD2 and its ligand CD58 is increased on monocytes (9) and eosinophils (8) of asthma patients. To directly address whether CD2 regulates allergic airway inflammation, we generated *Cd2*^−/−^ mice on Balb/c background. Important for our studies, these animals completely lack the surface expression of CD2 similar to *Cd2*^−/−^ mice on the C57BL/6 (B6) background (B6. *Cd2*^−/−^, Supplemental Figure 1) yet retain the normal incidence and numbers of innate and adaptive immune cell populations in various organs (Supplemental Figure 2 and *data not shown*).

Several costimulatory molecules play a role in asthma pathogenesis (4). With the exception of CD2, basal expression of several costimulatory molecules and their ligands were similar in both the WT and *Cd2*^−/−^ mice (Figure 1B). Employing a murine model of human asthma, which involves the use of HDME (Figure 1C), we observed that CD2 but not CD48 mRNA expression was elevated in the lung tissues of mice sensitized with HDME as compared to control animals that received saline (Figure 1D), suggesting that CD2 is likely to regulate the allergic immune response in asthma. Flow cytometric analysis revealed that various immune cells in the lungs have high basal expression of CD2 (Figure 1E), which was slightly increased on dendtric cells (DC) and macrophages (Mφ), while it remained largely unaltered in T, B natural killer (NK) and invariant NKT cells following HDME treatment (Figures 1E,F). However, there was an increased influx of various CD2-bearing immune cell populations in the lungs following allergen exposure. Specifically, a 1.9 ± 0.23, 2.0 ± 0.35, 2.5 ± 0.32 and 5.6 ± 1.6 fold increase was observed in CD4 T, CD8 T, macrophage and DC cell counts, respectively, as compared to cell numbers in the lungs of PBS-control mice.

### CD2 Regulates AHR but Not IgE Production in HDME-Induced Allergic Asthma

**Figure 2:**
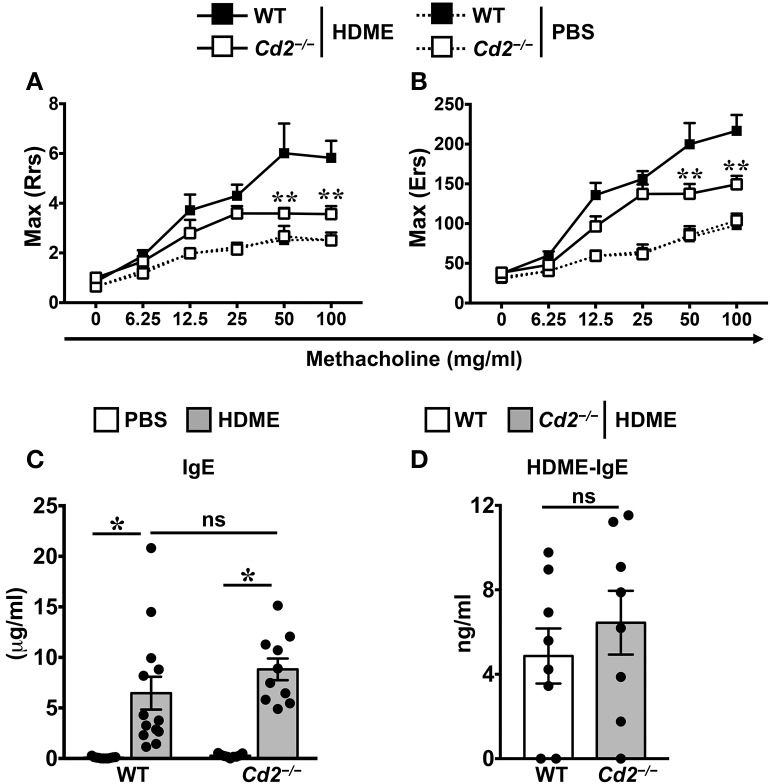
CD2 regulates AHR but not IgE production in HDME-induced mouse model of asthma. **(A,B)** WT and *Cd2*^−/−^ mice that were either injected with PBS or HDME were anesthetized 24 h after the last HDME challenge and analyzed for AHR. Assessment of lung constriction (Resistance [Rrs], **A**) and elastic stiffness of lungs, chest walls, and airways (Elastance [Ers], **B**) after challenge with increasing doses of methacholine. **(C,D)** Levels of serum IgE **(C)** and HDME-specific IgE **(D)** in WT and *Cd2*^−/−^ mice treated with PBS or challenged with HMDE. Data is shown as mean ± SEM and pooled from 3 to 4 independent experiments with a total of 8–13 mice per cohort. Statistical significance was determined by two-way ANOVA **(A,B)** and Student's unpaired *t*-test with Welch's correction **(C,D)**. **p ≤ 0.01, *p ≤ 0.05.

To assess the role of CD2 in allergic asthma, PBS-control and HDME-treated WT and *Cd2*^−/−^ mice were anesthetized and AHR parameters were assessed in response to methacholine (Mch) challenge. Baseline central airway resistance (Rrs) and lung elastance (Ers) were virtually identical in the WT and *Cd2*^−/−^ mice injected with PBS (Figures 2A,B). Compared to PBS controls, HDME-exposed WT mice demonstrated significant elevation in Rrs and Ers measurements, in a dose-dependent manner over a range of 6.25–100 mg/ml of Mch. In contrast, HDME-treated *Cd2*^−/−^ mice manifested a decreased AHR compared to similarly treated WT mice (Figures 2A,B), suggesting that CD2 regulates AHR in HDME-induced asthma.

Allergic asthma is characterized by elevated levels of serum IgE (3). Accordingly, we observed that serum IgE was significantly higher in HDME-treated WT and *Cd2*^−/−^ mice as compared to their respective saline controls (Figure 2C). HDME-induced total serum IgE (Figure 2C) as well as HDME-specific IgE (Figure 2D) levels were comparable in the allergen-treated WT and *Cd2*^−/−^ mice, indicating that CD2 does not regulate IgE production after allergen exposure.

### CD2 Regulates Lung Inflammation in an HDME-Induced AHR Model

**Figure 3:**
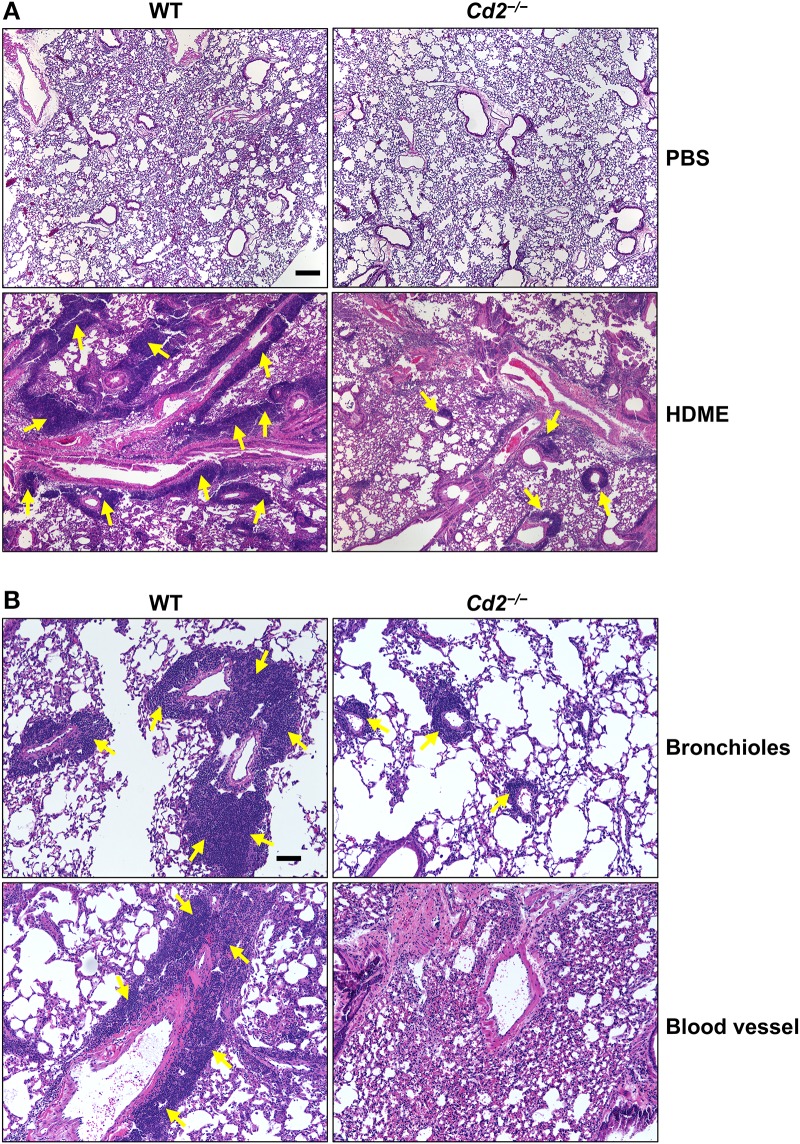
*Cd2*^−/−^ mice have reduced HDME-induced lung inflammation. Hematoxylin and eosin (H&E) staining of lung section of WT and *Cd2*^−/−^ mice injected with PBS or challenged with HMDE. Scale bar: 200 μm. **(A)** Representative micrographs of the lung sections of PBS-injected (top panels) and HDME-treated (lower panels) WT and *Cd2*^−/−^ mice at 4X magnification. **(B)** Representative images of bronchioles (top panels) and blood vessels (lower panels) from WT and *Cd2*^−/−^ mice challenged with HDME, at 10X magnification. Yellow arrows indicate cellular infiltration (inflammation). Data is representative of 3 independent experiments with a total of 8–13 mice per cohort.

**Figure 4:**
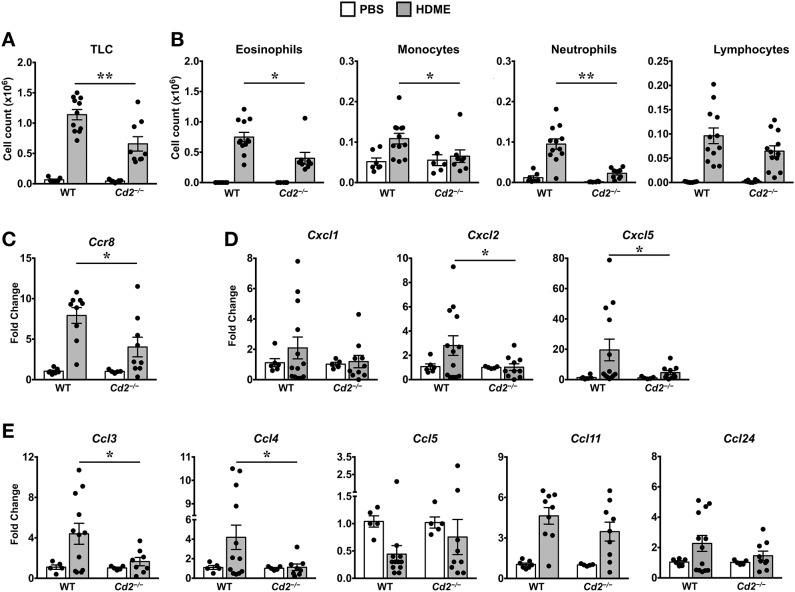
Reduced cellular infiltration and chemokine expression in *Cd2*^−/−^ mice challenged with HDME. **(A,B)** BALF from WT and *Cd2*^−/−^ mice injected with PBS or challenged with HMDE were analyzed for total leukocyte counts (TLC, **A**) and diffential cell counts (showing total monocytes, eosinophils, monocytes, neutrophils, and lymphocytes, **B**). Data is shown as mean ± SEM and pooled from 4 independent experiments with a total of 10–16 mice per cohort. **(C,D)** Lung tissue homogenates of WT and *Cd2*^−/−^ mice injected with PBS or HMDE were analyzed for mRNA levels of *Ccr8* **(C)** and various neutrophil **(D)** and eosinophil chemokines **(E)** by qPCR. Data is shown as mean fold change ± SEM and pooled from 3 independent experiments with a total of 8–13 mice per cohort. Statistical significance was determined by Student's unpaired *t*-test with Welch's correction. *p ≤ 0.05, **p ≤ 0.01.

Acute exposure to allergen increases AHR that is often associated with airway inflammation characterized by an influx of inflammatory cells (3). Having observed that CD2 regulates HDME-induced AHR and that there is an increased infiltration of CD2-bearing immune cells in the lungs of HDME-exposed mice (Figures 1D–F), we next determined if CD2 promotes lung inflammation. Histological analysis of the lungs revealed that while HDME induced lung inflammation in WT mice, it was substantially attenuated in *Cd2*^−/−^ mice (Figure 3A), which was more evident around the peribronchial and perivascular areas of the lung tissue sections (Figure 3B). To determine whether the decreased lung pathology observed in the absence of CD2 was due to a reduction in the recruitment of inflammatory cells to the airways, we harvested BALF and analyzed for total (Figure 4A) and differential inflammatory cell counts (Figure 4B). Following exposure to HDME, an acute inflammation was seen in the BALF of WT mice. The inflammatory infiltrate consisted of >80% eosinophils along with lower percentages of monocytes, lymphocytes and neutrophils. Consistent with lung histology, *Cd2*^−/−^ HDME mice exhibited reduced total BALF cell counts (Figure 4A). Specifically, monocyte, eosinophil and neutrophil counts were all lower in the BALF of *Cd2*^−/−^ HDME mice, with no change in lymphocyte counts (Figure 4B). Further analysis of chemokines and chemokine receptors revealed that while gene expression of CCR8 (Figure 4C), CXCL2, CXCL5 (Figure 4D), CCL3 and CCL4 (Figure 4E) were significantly reduced in *Cd2*^−/−^ HDME mice, mRNA levels of CXCL1, CCL5, CCL11, and CCL24 were comparable in HDME-treated WT and *Cd2*^−/−^ mice (Figures 4D,E).

### CD2 Promotes Asthma Pathogenesis via IL-13 Regulation

**Figure 5:**
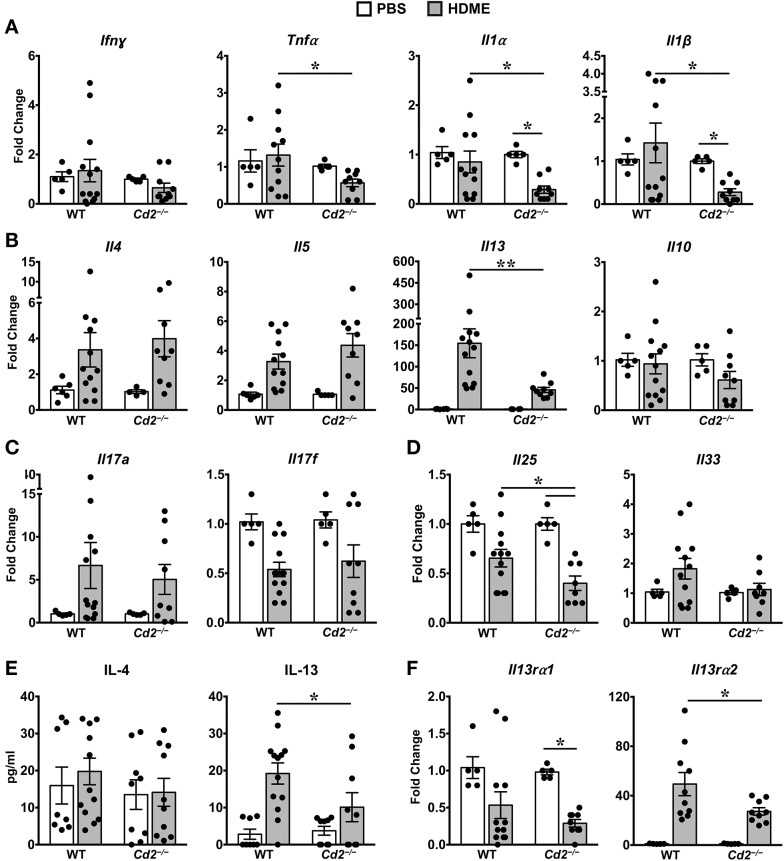
IL-13 and IL-13 receptor expression is significantly reduced in the lungs of HDME-challenged *Cd2*^−/−^ mice. **(A–D)** Lungs of WT and *Cd2*^−/−^ mice injected with PBS or HMDE were analyzed for gene expression of **(A)** Th1 (*Ifn*γ) and pro-inflammatory cytokines (*Tnf*α*, Il1*α and *Il1*β), **(B)** Th2 cytokines (*Il4, Il5, Il10* and *Il13*), **(C)** IL-17 family of cytokines (*Il17a* and *Il17f*), and **(D)** Alarmins (*Il25, Il33*). **(E)** IL-4 and IL-13 cytokine levels in the BALF supernatant of WT and *Cd2*^−/−^ mice challenged or not with HMDE. **(F)** Lungs of WT and *Cd2*^−/−^ mice injected with PBS or HMDE were analyzed for gene expression of IL-13 receptors *(Il13ra1 and Il13ra2)*. Data is presented as mean ± SEM and is pooled from 3 independent experiments with a total of 8–13 mice per cohort. Statistical significance was determined by using Student's unpaired *t*-test with Welch's correction. *p ≤ 0.05, **p ≤ 0.01.

Th1 cytokines can counter regulate Th2 cytokine expression and vice versa (10, 11). However, we observed that gene expression of Th1 cytokine such as IFNγ and other pro-inflammatory cytokines such IL-1α, IL-1β, and TNFα were comparable in PBS and HDME-treated WT mice (Figure 5A). In contrast, gene expression of these pro-inflammatory cytokines (except for IFNγ) were significantly decreased in *Cd2*^−/−^ HDME mice when compared to PBS controls or WT HDME mice (Figure 5A). As HDME manifest a Th2-high endotype (12), we next analyzed Th2 cytokines in the lung tissues of allergen-exposed WT and *Cd2*^−/−^ mice (Figure 5B). We observed HDME-induced increase in the mRNA levels of IL-4, IL-5, and IL-13 in the lungs of both WT and *Cd2*^−/−^ mice. However, the most dramatic increase was observed for IL-13 (155 ± 33 fold change) that was significantly reduced in *Cd2*^−/−^ HDME mice (45 ± 6, fold change, Figure 5B). Since only IL-13 (but not IL-4 or IL-5) expression was diminished in *Cd2*^−/−^ HDME mice, our data demonstrate a specific regulation of IL-13 by CD2 in asthma. In contrast, IL-10 levels were not affected by HDME or absence of CD2 (Figure 5B).

Recent evidence suggests that cytokines of the IL-17 family are also increased in BALF samples from patients with asthma (13). However, we observed little differences in IL-17A and IL-17F levels of HDME-exposed WT and *Cd2*^−/−^ mice (Figure 5C). Furthermore, epithelial cell-derived cytokines such as IL-33 and IL-25 (also known as alarmins) can play major role in the initiation of Th2-high asthma (14). We observed a significant increase in IL-33 levels in HDME treated WT mice that tended to be reduced in *Cd2*^−/−^ HDME mice. (Figure 5D). In contrast, IL-25 was significantly reduced in HDME treated *Cd2*^−/−^ mice (Figure 5D). This is particularly interesting because studies have shown that IL-25 can regulate the local production of IL-13 in the lungs (15). Consistent with the gene expression data, we also observed a reduction only in IL-13 but not IL-4 protein levels in the BALF of *Cd2*^−/−^ HDME mice when compared to WT allergen-challenged mice (Figure 5E). Taken together, our studies reveal a previously undefined role of CD2 in the regulation of IL-13.

### CD2 Regulates IL-13Rα1 and IL-13Rα2 Expression in the Lungs of Allergen-Treated Mice

IL-13 signaling occurs through IL-4Rα and IL-13Rα1 heterodimer to promote Th2 immunity (16) and IL-13 signaling induces IL-13Rα2 expression (17). Evidence suggests that IL-13Rα1 and IL-13Rα2 can modulate HDME-induced AHR, lung inflammation and mucus production (17–20). As CD2 specifically affected HDME-induced IL-13 production, AHR and lung inflammation, we analyzed IL-13Rα1 and IL-13Rα2 gene expression in the lungs of WT and *Cd2*^−/−^ mice challenged with HDME (Figure 5F). We observed that basal IL-13Rα1 expression was comparable in WT and *Cd2*^−/−^ mice, however, following HDME treatment, IL-13Rα1 expression was downregulated only in *Cd2*^−/−^ mice. In contrast to IL-13Rα1, IL-13Rα2 was robustly increased in response to HDME, in both cohorts, but the expression of IL-13Rα2 was dramatically decreased in *Cd2*^−/−^ HDME mice (27.2 ± 2.9 fold change), relative to WT allergen-treated mice (49.4 ± 9.4 fold change). Thus, it is likely that CD2 regulates the pathogenesis of HDME-induced AHR and inflammation via the regulation of IL-13Rα1 and IL-13Rα2.

### CD2 Regulates HDME-Induced Gene Expression of Polymeric Mucins and Goblet Cell Hyperplasia

**Figure 6:**
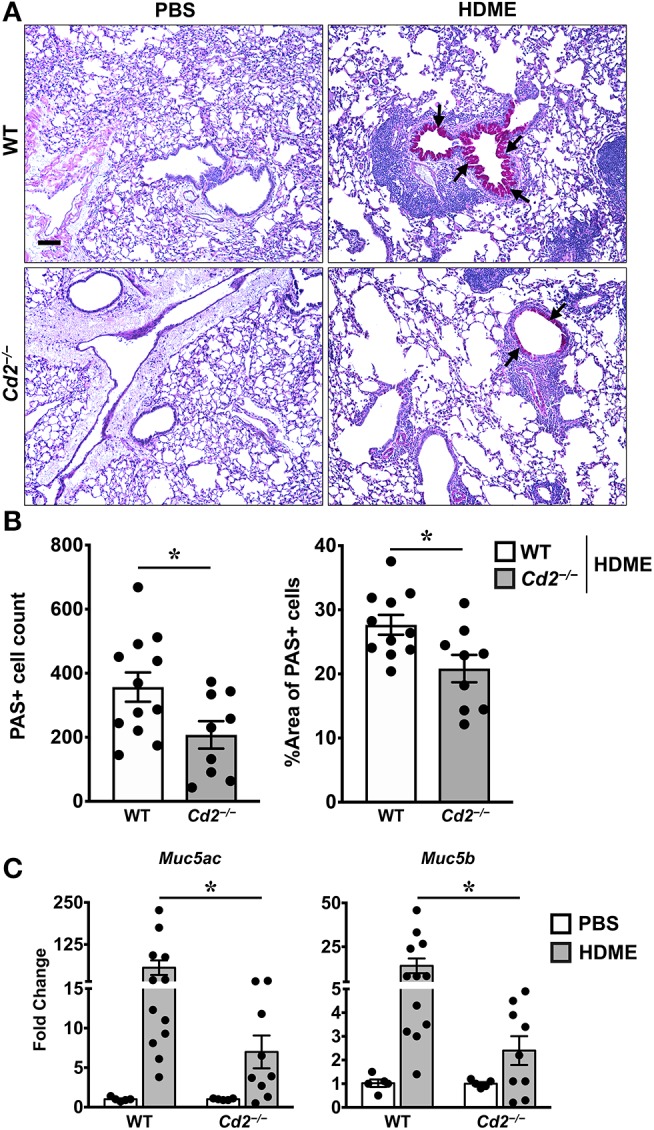
Reduced goblet cell hyperplasia and mucin production in the lungs of HDME-challenged *Cd2*^−/−^ mice. WT and *Cd2*^−/−^ mice were challenged with or without HMDE. Twenty-four hours after the last injection of HDME, mice were sacrificed and lung tissues were analyzed for PAS staining **(A,B)** and gene expression **(C)** of polymeric mucins (*Mus5ac* and *Muc5b*). Representative lung images **(A)** and PAS positive cell counts and % area of PAS positive cells **(B)** are shown. Scale bar: 200 μm. Data in B, C are presented as mean ± SEM and are pooled from 3 independent experiments with a total of 10–12 mice per cohort **(A,B)** and 8–13 mice per cohort in **(C)**. Statistical significance was determined using Student's unpaired *t*-test with Welch's correction. *p ≤ 0.05.

The direct downstream target of IL-13 is mucus production by goblet cells in the airways (21). Increased numbers of goblet cells (goblet cell hyperplasia) in part contributes to airway remodeling in asthma that evokes mucus hypersecretion and airflow obstruction (3). We analyzed goblet cell hyperplasia through PAS staining of lung sections from WT and *Cd2*^−/−^ HDME treated mice. Consistent with the diminished IL-13 expression, HDME-treated *Cd2*^−/−^ mice exhibited reduced PAS staining (Figure 6A). We also quantified PAS+ cell count and percent area of PAS+ staining and observed a decrease in these parameters in the HDME-challenged *Cd2*^−/−^ mice (Figure 6B).

In mice, excess mucus production is a cardinal feature of asthma that is regulated by 2 polymeric mucins: Muc5ac and Muc5b (22). We observed that both *Muc5ac* and *Muc5b* gene transcripts were significantly upregulated in the lungs of HDME-treated mice and consistent with prior studies (22), expression of *Muc5ac* was more than *Muc5b* in these animals (Figure 6C). In agreement with the PAS histology data (Figure 6A), mRNA levels of Muc5ac and Muc5b were decreased in HDME-treated *Cd2*^−/−^ mice. As these genes are direct downstream targets of IL-13, our data suggests that CD2 promotes goblet cell hyperplasia via IL-13 that in turn regulates these polymeric mucins.

### CD2 Regulates Various miRNA Expression in Asthma

**Figure 7:**
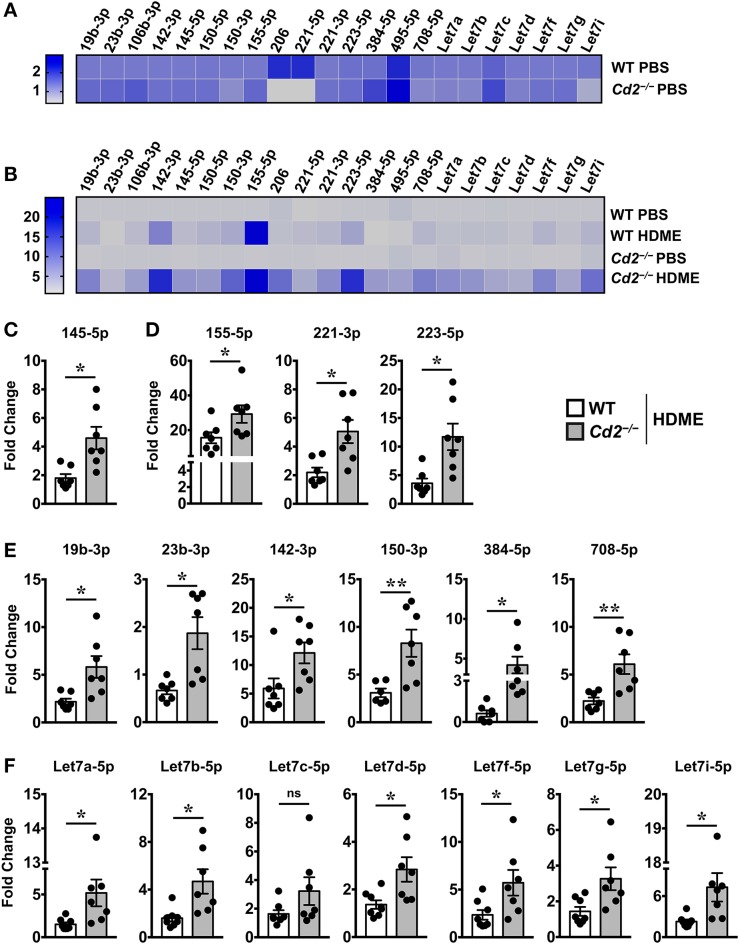
Increased expression of various miRNAs in the lungs of *Cd2*^−/−^ mice challenged with HDME. Lungs tissues of PBS-injected or HDME-treated WT and *Cd2*^−/−^ mice were analyzed for gene expression of various miRNAs as indicated in the figure. **(A)** Heat map of gene expression of various miRNAs in lungs of PBS-treated *Cd2*^−/−^ mice normalized to similarly treated WT animals. **(B)** Heat map of gene expression of various miRNAs in the lungs of WT and *Cd2*^−/−^ mice injected with HDME, normalized to respective PBS control mice. **(C–E)** Fold change in expression of various miRNAs regulating mucin production **(C)**, inflammation **(D)**, airway smooth muscle proliferation and AHR **(E)** and IL-13 production **(F)** in the lungs of WT and *Cd2*^−/−^ mice that were injected with HMDE. Fold change for each genotype was normalized to respective PBS control mice. Data is presented as mean ± SEM and are pooled from 2 independent experiments with a total of 7–8 mice per cohort. Statistical significance was determined using Student's unpaired *t*-test with Welch's correction. *p ≤ 0.05 or **p ≤ 0.01.

Several microRNAs (miRNAs) play either a protective or proinflammatory role in asthma (23). As depicted in the heat map, we screened for various miRNAs that have known roles in different aspects of asthma pathogenesis (Figures 7A,B). Both WT and *Cd2*^−/−^ PBS controls had comparable levels of most miRNAs analyzed (Figure 7A) indicating that the absence of CD2 did not alter the basal miRNA expression in the lungs. Furthermore, most of these miRNAs with the exception of miR-23b-3p, were increased in WT HDME mice in comparison to their PBS controls (Figure 7B). Strikingly, in comparison to allergen-treated WT mice, *Cd2*^−/−^ HDME-treated mice had higher expression of miRNAs that regulate polymeric mucin expression (24) (Figure 7C), inflammation (25–27) (Figure 7D), airway smooth muscle proliferation and AHR (28–33) (Figure 7E). Recently it has been shown that Let-7 family of miRNAs can directly bind to the 3′ UTR of IL-13 mRNA and degrade it (34). Consistently, we observed that with the exception of Let-7c, all the Let-7 family of miRNAs were significantly increased in the *Cd2*^−/−^ HDME mice in comparison to similarly treated WT animals (Figure 7F). Thus, our data suggest that in the absence of CD2, IL-13 gene expression is suppressed at least in part by Let-7 family of miRNAs.

## Discussion

CD2 is expressed on several hematopoietic cells including T and B lymphocytes, natural killer cells, macrophages/monocytes and dendritic cells (35). Previous studies have shown that a subset of CD2^high^ monocytes expressing FcεRI correlate with higher serum IgE levels (9) in asthma patients. Indeed, we observed higher CD2 gene expression in the lungs of human asthmatics as compared to healthy donors. Consistently, we also observed increased CD2 mRNA levels in the lungs of HDME-exposed WT mice which was largely due to increased infiltration of several CD2-bearing immune cells into the lungs. Our results are consistent with a prior study where CD2 expression was upregulated in ovalbumin (OVA)- and *Aspergillus*-exposed asthmatic mice, although the increase was more pronounced in the OVA model (36). In the same study, the authors demonstrate that CD48, the ligand for CD2 was robustly upregulated in both the models. However, contrary to this study (36), we did not observe any allergen-induced change in CD48 expression. These discrepancies highlight that depending on the allergen and the sensitization protocol, the physiological outcomes and immune responses are likely to vary. For the current study, we used a HDME-challenged murine model of asthma as HDM is associated with allergic responses in up to 85% of asthma patients worldwide (37, 38). Furthermore, HDME is a naturally-inhaled antigen and repeated exposure to this allergen does not induce tolerance (12).

A striking finding of our study is that CD2 promotes lung inflammation, AHR and goblet cell hyperplasia that is specifically regulated by IL-13 levels. Indeed, several studies support the notion that IL-13 is the major effector molecule in asthma (21, 39). IL-13 regulates AHR by inducing airway smooth muscle cell (ASMC) proliferation and contraction (40, 41). Additionally, IL-13 may increase AHR by augmenting mucus secretion that results in thickening of the airways (42, 43). In line with this notion, transgenic mice overexpressing IL-13 have increased mucus production (42–44) whereas *Il13*^−/−^ mice have a marked reduction in mucus secretion (45). Moreover, while administration of IL-13 into the lungs of WT mice induces mucus production (39, 46), neutralization of IL-13 reverses this effect (39). Consistent with these observations, we demonstrate that polymeric mucins, Muc5ac and Muc5b, that primarily regulate mucus production in mice (22) are significantly reduced in allergen-treated *Cd2*^−/−^ mice. Thus, our studies reveal that CD2 regulates goblet cell hyperplasia and mucus overproduction by modulating IL-13 and its downstream targets Muc5ac and Muc5b.

IL-13 also modulates serum IgE levels as transgenic mice overexpressing IL-13 have higher (43, 44) and *Il13*^−/−^ mice have reduced serum IgE (47). However, we did not observe any decrease in serum IgE levels in *Cd2*^−/−^ HDME mice. Consistent with our data, blockade of IL-13 by systemic administration of a soluble IL-13Rα2-IgG Fc fusion protein results in diminished OVA-induced AHR and mucus hypersecretion but has no impact on allergen-specific IgE levels (39). It is well-established that B cell differentiation and IgE production is primarily controlled by IL-4 (14). Thus, the comparable levels of IgE in HDME-exposed WT and *Cd2*^−/−^ mice can be attributed to similar expression of IL-4 in these mice. Indeed, a prior study supports that IL-13 facilitates IgE production, particularly in the absence of IL-4 (48). Although IL-4 and IL-13 share many functional activities, it is believed that IL-13 is the central mediator of Th2-dominant immune responses (16), which is likely due to its greater production and persistence. Consistent with this notion, IL-13 is produced 100-fold greater than IL-4 and sustains for a longer period in a murine model of fibrosis induced by *Schistosoma mansoni* infection (17). Similarly, we observe that IL-13 is dramatically higher than IL-4 in the lungs and BALF of WT asthmatic mice. Given that CD2 regulates only IL-13 but not IL-4, our studies indicate that CD2 promotes an IL-13-dominant induction of Th2-allergic responses.

Airway eosinophilia is a characteristic feature of Th2-high allergic asthma (1). IL-5 is a key cytokine for the differentiation, activation and survival of eosinophils and together with IL-4 and IL-13, it regulates the recruitment of these cells into the lungs (14). As both IL-4, and IL-5 mRNA levels are unaffected by CD2 deficiency, yet eosinophilia was greatly reduced in HDME-treated *Cd2*^−/−^ mice, our observations highlight the importance of IL-13 as the central regulator of HDME-induced lung inflammation. This is further supported by the ability of IL-13 to induce VCAM-1 expression on endothelial cells, which results in recruitment of T cells, monocytes and eosinophils to the sites of allergic inflammation (49). Additionally, several chemokines, particularly CCL5, CCL11, and CCL24 are known to regulate eosinophil trafficking to the lungs (50, 51). Surprisingly, in our model, HDME failed to upregulate CCL5, but increased expression of CCL11and CCL24, as well as other eosinophil recruiters CCL3 (MIP-1α) and CCL4 (MIP-1β) (50, 51). Interestingly, while CCL3 and CCL4 levels were decreased in allergen-exposed *Cd2*^−/−^ mice, the expression of CCL11 and CCL24 were unaltered in these mice. Given that CCL3 regulates initial eosinophil localization whereas CCL11 plays a role in secondary eosinophil infiltration and degranulation (52), our data suggest that CD2 regulates early events in allergic eosinophilia. We demonstrate that CD2 also regulates monocyte and neutrophil infiltration into asthmatic lungs. This is consistent with reduced IL-13 (chemoattractant for monocytes) (53), CCL3 and CCL4 (recruit monocytes and neutrophils) (50, 51) as well as CXCL2 and CXCL5 (recruit neutrophils) (50, 51) levels in HDME-treated *Cd2*^−/−^ mice.

CCR8 is expressed on regulatory T helper cells, CD4^+^ thymocytes, a subset of dendritic cells, macrophages and Th2 (but not Th1) cells (54, 55). In a previous study (56), it was demonstrated that the CCL8-CCR8 chemokine axis is a crucial regulator of Th2 cell homing that drives IL-5–mediated chronic allergic inflammation. Paradoxically, although the chemokine receptor CCR8, was decreased in allergen-exposed *Cd2*^−/−^ mice, total lymphocyte numbers were intact. Given the redundancy of chemokine and chemokine receptor functions in leukocyte trafficking, it is tempting to speculate that other chemokine receptors are normally expressed on Th2 cells of HDME-treated *Cd2*^−/−^ mice. This would be also consistent with normal levels of IL-4 and IL-5 in these mice.

Although IL-13 is primarily produced by Th2 cells (14), tissue eosinophils can also rapidly produce bioactive IL-13 following activation with cytokines or CD28-ligation (57, 58). As eosinophils outnumber T cells in the lungs of Th2 asthmatic mice, the presence of eosinophils is an important determinant for local tissue IL-13 levels. Accordingly, reduced IL-13 in the lungs of HDME-exposed *Cd2*^−/−^ mice can be attributed, at least in part, to the reduced eosinophil numbers. IL-13 mediates its effector functions via a receptor complex that includes IL-4Rα, IL-13Rα1 and IL-13Rα2. Although prior studies have demonstrated that both IL-13Rα1 and IL-13Rα2 regulate AHR and mucus production (17–20), the high affinity IL-13Rα2 but not IL-13Rα1 is induced by HDME. Interestingly, IL-13Rα2 and its ligand IL-13 cross-regulate each other (17) which is in line with our data that both IL-13 and IL-13Rα2 are decreased in HDME-exposed *Cd2*^−/−^ mice. Thus, our studies implicate that CD2 regulates the expression of IL-13 and its receptors, however, the exact mechanism remains to be elucidated.

Epithelial-derived cytokines such as thymic stromal lymphopoietin (TSLP), IL-25 and IL-33 are important factors in the pathogenesis of asthma as they promote Th2-associated immune responses via activation of DCs (14). Alternatively, these epithelial cytokines can induce activation and terminal differentiation of effector Th2 cells, innate lymphoid cells and other innate immune cells such as basophils, macrophages and eosinophils (14). While we observed a significant increase in IL-33 in HDME-treated Balb/c mice, gene expression of IL-25 or TSLP (data not shown) were unaltered in these mice. Similarly, proinflammatory cytokines such as IL-1α, IL-1β, and TNF-α, also produced by epithelial cells were not affected by HDME exposure in Balb/c mice. However, these epithelial cytokines (IL-25, IL-1α, IL-1β, and TNF-α) were significantly downregulated in HDME-exposed *Cd2*^−/−^ mice. Interestingly, in an experimental mouse model of chronic lung disease after viral infection (59), activation of NKT cell-macrophage immune axis leads to increased expression of IL-13R and production of IL-13 that drives a positive feedback loop to amplify IL-13 production and differentiation of airway epithelial cells and ASMC to become hyperreactive. Thus, while it is unclear how CD2 regulates airway epithelial cells, it is likely that reduced IL-13 production and lower IL-13R expression in HDME-treated *Cd2*^−/−^ mice dampens cytokine production by airway epithelial cells and ameliorates AHR in our model.

Recent studies suggest that miRNAs modulate multiple aspects of asthma pathogenesis (23). For example, increased miR-145-5p downregulates Muc5ac expression in an OVA-induced model of allergy (24). MiR-155-5p, has been implicated to play varied roles in asthma in many different cell types (25) and can directly target IL-13Rα1 mRNA preventing translation into protein (27). Similarly, miR-223 inversely regulates eosinophil and neutrophil progenitors (24, 26). Furthermore, increased expression of several miRNAs inhibit ASMC proliferation or AHR via different pathways. Specifically, miR-19b-3p, miR23b-3p and miR-142-3p inhibit TGFβ-dependent ASMC proliferation (28–30). MiR-150-3p and miR-708-5p inhibit ASMC proliferation by downregulating BCYRN1 (31) and CD38 (33), respectively, while miR-384-5p reduces ASMC autophagy (32). Strikingly, all these miRNAs were increased in HDME-treated *Cd2*^−/−^ mice. Additionally, most Let-7 family of miRNAs were upregulated in these mice. Let-7 miRNAs can directly target IL-13 transcripts and intranasal delivery of Let-7 miRNA attenuates allergen-induced AHR in OVA-treated mice (34). Thus, it is plausible that increased Let-7 miRNAs downregulate IL-13, that ameliorates AHR and inflammation in HDME-treated *Cd2*^−/−^ mice. Collectively, these data provide a novel mechanistic insight into the regulation of IL-13, mucus production, lung inflammation and AHR by CD2. However, the exact mechanism by which CD2 regulates the expression of various miRNAs in specific immune cell types, that in turn, control HDME-induced allergic reponse warrants further investigation.

Although IL-13 regulates a wide spectrum of allergic response in murine models of asthma, anti-IL-13 therapy has not been successful in clinical trials suggesting that blocking IL-13 alone is not effective (60). As IL-13 and IL-13 receptors cross-regulate each other, an alternative approach could be to block both IL-13 and its receptors simultaneously. Recent studies have shown that the combined blockade of IL-13 and IL-33 (61) or IL-13 and IL-25 (62) leads to greater inhibition of allergic reponse than suppressing the activity of a single cytokine. While the role of CD2 in human lung disease remains largely unknown, our data are the first to demonstrate that genetic ablation of CD2 in mice results in significant reduction in AHR, lung inflammation and goblet cell hyperplasia that is specifically associated with the expression of IL-13 and its receptors. Consistently, in a prior study, antibody blockade of CD2 or CD48 (ligand for CD2) abrogated OVA-induced lung inflammation, ASMC thickening, goblet cell hyperplasia and mucus production (36). As CD2 is primarily expressed on immune cells, but impacts functions of airway epithelial cells and AMSCs as well as expression of several miRNAs, our data provide a foundation to explore the mechanistic basis of HDME-induced immunopathology with the potential to develop novel therapies for allergic asthma.

## Data Availability Statement

All datasets generated for this study are included in the article/Supplementary Material.

## Ethics Statement

The animal study was reviewed and approved by Michigan State University IACUC.

## Author Contributions

TH performed experiments and analyzed data. AK performed experiments and assisted with AHR data analysis. KT, RG, and KM performed FACS staining, ELISA, qPCR (human lung samples) respectively. RP provided lung samples and edited the manuscript. HS assisted with AHR studies and wrote parts of the manuscript. RD designed and supervised the research, analyzed the data, and wrote the manuscript.

## Conflict of Interest

The authors declare that the research was conducted in the absence of any commercial or financial relationships that could be construed as a potential conflict of interest.

## Supplementary Material

Supplemental Figure 1 Immunophenotyping of *Cd2^−/−^* mice on Balb/c background. **(A,B)** Peripheral blood mononuclear cells (PBMCs) from C57BL/6 (B6), B6. *Cd2^−/−^*, Balb/c and Balb/c. Peripheral blood mononuclear cells (PBMCs) from C57BL/6 (B6), B6. *Cd2^−/−^*, Balb/c, and Balb/c. *Cd2^−/−^* mice were analyzed for surface expression of **(A)** CD2 and TCRβ and **(B)** H2k^b^ and H2k^d^ by flow cytometry. Representative density plots from at least 4 mice per genotype are shown.

Supplemental Figure 2 *Cd2^−/−^* mice have normal immune cell distribution. **(A,B)** Representative flow cytometry analysis showing various lymphocyte (B, NK, iNKT, T, CD4 T, CD8 T cell) populations **(A)**, and gating strategy for innate cells **(B)** in lungs of naïve WT mice, as indicated. **(C,D)** Absolute numbers of lymphocytes **(C)** and innate cells **(D)** in lungs of naïve WT and *Cd2^−/−^* mice. Data shown is representative of 4 mice for each genotype. Statistical significance was determined by Student's *t* test with Welch's correction. **p* ≤ 0.05, ***p* ≤ 0.01.

Supplemental Table 1 Asthma patient and normal donor information.

Supplemental Table 2 Antibodies used for flow cytometry.

## References

[1] Holgate ST. Innate and adaptive immune responses in asthma. Nat Med. (2012) 18:673–83. 10.1038/nm.2731 22561831

[2] Russell RJ Brightling C. Pathogenesis of asthma: implications for precision medicine. Clin Sci (Lond). (2017) 131:1723–35. 10.1042/CS20160253 28667070

[3] Lambrecht BN Hammad H. The immunology of asthma. Nat Immunol. (2015) 16:45–56. 10.1038/ni.3049 25521684

[4] Lombardi V Singh AK Akbari O. The role of costimulatory molecules in allergic disease and asthma. Int Arch Allergy Immunol. (2010) 151:179–89. 10.1159/000242355 19786798 PMC2837887

[5] Hahn WC Menu E Bothwell AL Sims PJ Bierer BE. Overlapping but nonidentical binding sites on CD2 for CD58 and a second ligand CD59. Science. (1992) 256:1805–7. 10.1126/science.1377404 1377404

[6] Green JM Karpitskiy V Kimzey SL Shaw AS. Coordinate regulation of T cell activation by CD2 and CD28. J Immunol. (2000) 164:3591–5. 10.4049/jimmunol.164.7.3591 10725714

[7] Skanland SS Moltu K Berge T Aandahl EM Tasken K. T-cell co-stimulation through the CD2 and CD28 co-receptors induces distinct signalling responses. Biochem J. (2014) 460:399–410. 10.1042/BJ20140040 24665965

[8] Walker C Braun RK Boer C Kroegel C Virchow JC Hansel TT. Cytokine control of eosinophils in pulmonary diseases. J Allergy Clin Immunol. (1994) 94:1262–71. 10.1016/0091-6749(94)90341-7 7798567

[9] Cheng YX Foster B Holland SM Klion AD Nutman TB Casale TB. CD2 identifies a monocyte subpopulation with immunoglobulin E-dependent, high-level expression of Fc epsilon RI. Clin Exp Allergy. (2006) 36:1436–45. 10.1111/j.1365-2222.2006.02578.x 17083354 PMC1661841

[10] Elser B Lohoff M Kock S Giaisi M Kirchhoff S Krammer PH. IFN-gamma represses IL-4 expression via IRF-1 and IRF-2. Immunity. (2002) 17:703–12. 10.1016/S1074-7613(02)00471-5 12479817

[11] Wurtz O Bajenoff M Guerder S. IL-4-mediated inhibition of IFN-gamma production by CD4+ T cells proceeds by several developmentally regulated mechanisms. Int Immunol. (2004) 16:501–8. 10.1093/intimm/dxh050 14978023

[12] Cates EC Fattouh R Wattie J Inman MD Goncharova S Coyle AJ. Intranasal exposure of mice to house dust mite elicits allergic airway inflammation via a GM-CSF-mediated mechanism. J Immunol. (2004) 173:6384–92. 10.4049/jimmunol.173.10.6384 15528378

[13] Irvin C Zafar I Good J Rollins D Christianson C Gorska MM. Increased frequency of dual-positive TH2/TH17 cells in bronchoalveolar lavage fluid characterizes a population of patients with severe asthma. J Allergy Clin Immunol. (2014) 134:1175–86. e1177. 10.1016/j.jaci.2014.05.038 25042748 PMC4254017

[14] Lambrecht BN Hammad H Fahy JV. The cytokines of asthma. Immunity. (2019) 50:975–91. 10.1016/j.immuni.2019.03.018 30995510

[15] Ballantyne SJ Barlow JL Jolin HE Nath P Williams AS Chung KF. Blocking IL-25 prevents airway hyperresponsiveness in allergic asthma. J Allergy Clin Immunol. (2007) 120:1324–31. 10.1016/j.jaci.2007.07.051 17889290

[16] Gour N Wills-Karp M. IL-4 and IL-13 signaling in allergic airway disease. Cytokine. (2015) 75:68–78. 10.1016/j.cyto.2015.05.014 26070934 PMC4532591

[17] Chiaramonte MG Mentink-Kane M Jacobson BA Cheever AW Whitters MJ Goad ME. Regulation and function of the interleukin 13 receptor alpha 2 during a T helper cell type 2-dominant immune response. J Exp Med. (2003) 197:687–701. 10.1084/jem.20020903 12642601 PMC2193852

[18] Daines MO Chen W Tabata Y Walker BA Gibson AM Masino JA. Allergen-dependent solubilization of IL-13 receptor alpha2 reveals a novel mechanism to regulate allergy. J Allergy Clin Immunol. (2007) 119:375–83. 10.1016/j.jaci.2006.09.039 17140645 PMC5472628

[19] Chen W Sivaprasad U Gibson AM Ericksen MB Cunningham CM Bass SA. Khurana Hershey K. IL-13 receptor alpha2 contributes to development of experimental allergic asthma. J Allergy Clin Immunol. (2013) 132:951–8. 10.1016/j.jaci.2013.04.016 23763980 PMC3836839

[20] Munitz A Brandt EB Mingler M Finkelman FD Rothenberg ME. Distinct roles for IL-13 and IL-4 via IL-13 receptor alpha1 and the type II IL-4 receptor in asthma pathogenesis. Proc Natl Acad Sci USA. (2008) 105:7240–45. 10.1073/pnas.0802465105 18480254 PMC2386078

[21] Wills-Karp M. Interleukin-13 in asthma pathogenesis. Immunol Rev. (2004) 202:175–90. 10.1111/j.0105-2896.2004.00215.x 15546393

[22] Bonser LR Erle DJ. Airway mucus asthma: the role of MUC5AC MUC5B. J Clin Med. (2017) 6:10. 10.20944/preprints201711.0010.v1 29186064 PMC5742801

[23] Lu TX Rothenberg ME. Diagnostic, functional, and therapeutic roles of microRNA in allergic diseases. J Allergy Clin Immunol. (2013) 132:3–13; quiz 14. 10.1016/j.jaci.2013.04.039 23735656 PMC3737592

[24] Cheng Z Dai LL Wang X Jia LQ Jing XG Li PF. MicroRNA-145 down-regulates mucin 5AC to alleviate airway remodeling and targets EGFR to inhibit cytokine expression. Oncotarget. (2017) 8:46312–25. 10.18632/oncotarget.17933 28564633 PMC5542269

[25] Zhou H Li J Gao P Wang Q Zhang J. miR-155: a novel target in allergic asthma. Int J Mol Sci. (2016) 17:17101773. 10.3390/ijms17101773 27783037 PMC5085797

[26] Johnnidis JB Harris MH Wheeler RT Stehling-Sun S Lam MH Kirak O. Regulation of progenitor cell proliferation and granulocyte function by microRNA-223. Nature. (2008) 451:1125–9. 10.1038/nature06607 18278031

[27] Martinez-Nunez RT Louafi F Sanchez-Elsner T. The interleukin 13 (IL-13) pathway in human macrophages is modulated by microRNA-155 via direct targeting of interleukin 13 receptor alpha1 (IL13Ralpha1). J Biol Chem. (2011) 286:1786–94. 10.1074/jbc.M110.169367 21097505 PMC3023473

[28] Zou M Wang F Gao R Wu J Ou Y Chen X. Autophagy inhibition of hsa-miR-19a-3p/19b-3p by targeting TGF-beta R II during TGF-beta1-induced fibrogenesis in human cardiac fibroblasts. Sci Rep. (2016) 6:24747. 10.1038/srep24747 27098600 PMC4838850

[29] Chen M Huang L Zhang W Shi J Lin X Lv Z. MiR-23b controls TGF-beta1 induced airway smooth muscle cell proliferation via TGFbetaR2/p-Smad3 signals. Mol Immunol. (2016) 70:84–93. 10.1016/j.molimm.2015.12.012 26748386

[30] Wang J Wang HS Su ZB. MicroRNA-142 inhibits proliferation and promotes apoptosis in airway smooth muscle cells during airway remodeling in asthmatic rats via the inhibition of TGF-beta -dependent EGFR signaling pathway. Cell Physiol Biochem. (2018) 47:1682–95. 10.1159/000490986 29949788

[31] Zhang XY Tang XY Ma LJ Guo YL Li XS Zhao LM. Schisandrin B down-regulated lncRNA BCYRN1 expression of airway smooth muscle cells by improving miR-150 expression to inhibit the proliferation and migration of ASMC in asthmatic rats. Cell Prolif. (2017) 50:12382. 10.1111/cpr.12382 28960519 PMC6529112

[32] Cheng Z Wang X Dai L Jia L Jing X Liu Y. Suppression of microRNA-384 enhances autophagy of airway smooth muscle cells in asthmatic mouse. Oncotarget. (2017) 8:67933–41. 10.18632/oncotarget.18913 28978085 PMC5620225

[33] Dileepan M Jude JA Rao SP Walseth TF Panettieri RA Subramanian S. MicroRNA-708 regulates CD38 expression through signaling pathways JNK MAP kinase and PTEN/AKT in human airway smooth muscle cells. Respir Res. (2014) 15:107. 10.1186/s12931-014-0107-0 25175907 PMC4156970

[34] Kumar M Ahmad T Sharma A Mabalirajan U Kulshreshtha A Agrawal A. Let-7 microRNA-mediated regulation of IL-13 and allergic airway inflammation. J Allergy Clin Immunol. (2011) 128:1077–1085.e1071–1010. 10.1016/j.jaci.2011.04.034 21616524

[35] Davis SJ Ikemizu S Wild MK van der Merwe PA. CD2 and the nature of protein interactions mediating cell-cell recognition. Immunol Rev. (1998) 163:217–36. 10.1111/j.1600-065X.1998.tb01199.x 9700513

[36] Munitz A Bachelet I Finkelman FD Rothenberg ME Levi-Schaffer F. CD48 is critically involved in allergic eosinophilic airway inflammation. Am J Respir Crit Care Med. (2007) 175:911–8. 10.1164/rccm.200605-695OC 17290046 PMC1899297

[37] Gandhi VD Davidson C Asaduzzaman M Nahirney D Vliagoftis H. House dust mite interactions with airway epithelium: role in allergic airway inflammation. Curr Allergy Asthma Rep. (2013) 13:262–70. 10.1007/s11882-013-0349-9 23585216

[38] Gregory LG Lloyd CM. Orchestrating house dust mite-associated allergy in the lung. Trends Immunol. (2011) 32:402–11. 10.1016/j.it.2011.06.006 21783420 PMC3381841

[39] Wills-Karp M Luyimbazi J Xu X Schofield B Neben TY Karp CL. Interleukin-13: central mediator of allergic asthma. Science. (1998) 282:2258–61. 10.1126/science.282.5397.2258 9856949

[40] Wei LH Jacobs AT Morris SM Jr Ignarro LJ. IL-4 and IL-13 upregulate arginase I expression by cAMP and JAK/STAT6 pathways in vascular smooth muscle cells. Am J Physiol Cell Physiol. (2000) 279:C248–56. 10.1152/ajpcell.2000.279.1.C248 10898736

[41] Tliba O Deshpande D Chen H Van Besien C Kannan M Panettieri RA. IL-13 enhances agonist-evoked calcium signals and contractile responses in airway smooth muscle. Br J Pharmacol. (2003) 140:1159–62. 10.1038/sj.bjp.0705558 14597600 PMC1574143

[42] Zhu Z Homer RJ Wang Z Chen Q Geba GP Wang J. Pulmonary expression of interleukin-13 causes inflammation, mucus hypersecretion, subepithelial fibrosis, physiologic abnormalities, and eotaxin production. J Clin Invest. (1999) 103:779–88. 10.1172/JCI5909 10079098 PMC408149

[43] Fallon PG Emson CL Smith P McKenzie AN. IL-13 overexpression predisposes to anaphylaxis following antigen sensitization. J Immunol. (2001) 166:2712–6. 10.4049/jimmunol.166.4.2712 11160336

[44] Emson CL Bell SE Jones A Wisden W McKenzie AN. Interleukin (IL)-4-independent induction of immunoglobulin (Ig)E, and perturbation of T cell development in transgenic mice expressing IL-13. J Exp Med. (1998) 188:399–404. 10.1084/jem.188.2.399 9670052 PMC2212457

[45] Walter DM McIntire JJ Berry G McKenzie AN Donaldson DD DeKruyff RH. Critical role for IL-13 in the development of allergen-induced airway hyperreactivity. J Immunol. (2001) 167:4668–75. 10.4049/jimmunol.167.8.4668 11591797

[46] Grunig G Warnock M Wakil AE Venkayya R Brombacher F Rennick DM. Requirement for IL-13 independently of IL-4 in experimental asthma. Science. (1998) 282:2261–63. 10.1126/science.282.5397.2261 9856950 PMC3897229

[47] McKenzie GJ Emson CL Bell SE Anderson S Fallon P Zurawski G. Impaired development of Th2 cells in IL-13-deficient mice. Immunity. (1998) 9:423–32. 10.1016/S1074-7613(00)80625-1 9768762

[48] Punnonen J Yssel H de Vries JE. The relative contribution of IL-4 and IL-13 to human IgE synthesis induced by activated CD4+ or CD8+ T cells. J Allergy Clin Immunol. (1997) 100:792–801. 10.1016/S0091-6749(97)70276-8 9438489

[49] Bochner BS Klunk DA Sterbinsky SA Coffman RL Schleimer RP. IL-13 selectively induces vascular cell adhesion molecule-1 expression in human endothelial cells. J Immunol. (1995) 154:799–803. 7529288

[50] Palmqvist C Wardlaw AJ Bradding P. Chemokines and their receptors as potential targets for the treatment of asthma. Br J Pharmacol. (2007) 151:725–36. 10.1038/sj.bjp.0707263 17471178 PMC2014125

[51] Pease JE. Asthma, allergy and chemokines. Curr Drug Targets. (2006) 7:3–12. 10.2174/138945006775270204 16454696

[52] Campbell EM Kunkel SL Strieter RM Lukacs NW. Temporal role of chemokines in a murine model of cockroach allergen-induced airway hyperreactivity and eosinophilia. J Immunol. (1998) 161:7047–53. 9862742

[53] Ip WK Wong CK Lam CW. Interleukin (IL)-4 and IL-13 up-regulate monocyte chemoattractant protein-1 expression in human bronchial epithelial cells: involvement of p38 mitogen-activated protein kinase, extracellular signal-regulated kinase 1/2 and Janus kinase-2 but not c-Jun NH2-terminal kinase 1/2 signalling pathways. Clin Exp Immunol. (2006) 145:162–72. 10.1111/j.1365-2249.2006.03085.x 16792687 PMC1942012

[54] Chensue SW Lukacs NW Yang TY Shang X Frait KA Kunkel SL. Aberrant *in vivo* T helper type 2 cell response and impaired eosinophil recruitment in CC chemokine receptor 8 knockout mice. J Exp Med. (2001) 193:573–84. 10.1084/jem.193.5.573 11238588 PMC2193397

[55] D'Ambrosio D Iellem A Bonecchi R Mazzeo D Sozzani S Mantovani A. Selective up-regulation of chemokine receptors CCR4 and CCR8 upon activation of polarized human type 2 Th cells. J Immunol. (1998) 161:5111–5. 9820476

[56] Islam SA Chang DS Colvin RA Byrne MH McCully ML Moser B. Mouse CCL8, a CCR8 agonist, promotes atopic dermatitis by recruiting IL-5+ T(H)2 cells. Nat Immunol. (2011) 12:167–77. 10.1038/ni.1984 21217759 PMC3863381

[57] Schmid-Grendelmeier P Altznauer F Fischer B Bizer C Straumann A Menz G. Eosinophils express functional IL-13 in eosinophilic inflammatory diseases. J Immunol. (2002) 169:1021–7. 10.4049/jimmunol.169.2.1021 12097410

[58] Woerly G Lacy P Younes AB Roger N Loiseau S Moqbel R. Human eosinophils express and release IL-13 following CD28-dependent activation. J Leukoc Biol. (2002) 72:769–79. 12377947

[59] Kim EY Battaile JT Patel AC You Y Agapov E Grayson MH. Persistent activation of an innate immune response translates respiratory viral infection into chronic lung disease. Nat Med. (2008) 14:633–40. 10.1038/nm1770 18488036 PMC2575848

[60] Marone G Granata F Pucino V Pecoraro A Heffler E Loffredo S. The intriguing role of interleukin 13 in the pathophysiology of asthma. Front Pharmacol. (2019) 10:1387. 10.3389/fphar.2019.01387 31866859 PMC6908970

[61] Ramirez-Carrozzi V Sambandam A Zhou M Yan D Kang J Wu X. Combined blockade of the IL-13 and IL-33 pathways leads to a greater inhibition of type 2 inflammation over inhibition of either pathway alone. J Allergy Clin Immunol. (2017) 139:705–8.e706. 10.1016/j.jaci.2016.08.026 27697499

[62] Zhang FQ Han XP Zhang F Ma X Xiang D Yang XM. Therapeutic efficacy of a co-blockade of IL-13 and IL-25 on airway inflammation and remodeling in a mouse model of asthma. Int Immunopharmacol. (2017) 46:133–40. 10.1016/j.intimp.2017.03.005 28282577

