# Supplementary figures and images for: CD2 Regulates Pathogenesis of Asthma Induced by House Dust Mice Extract
Source: Front Immunol. 2020 May 12;11:881. doi: 10.3389/fimmu.2020.00881 (PMC7235426; doi:10.3389/fimmu.2020.00881)

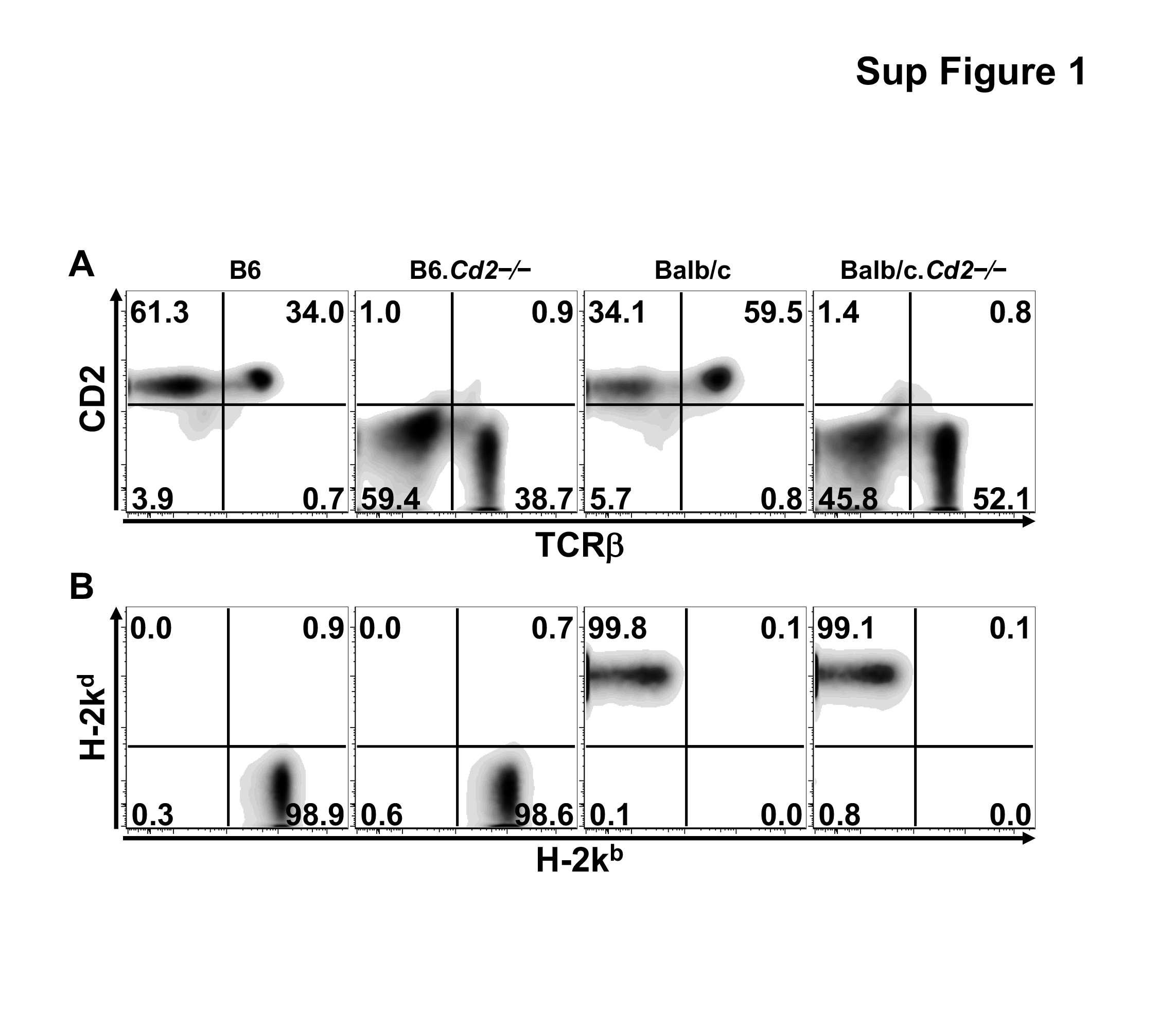

Supplement: Supplemental Figure 1 — Immunophenotyping of Cd2−/− mice on Balb/c background. (A,B) Peripheral blood mononuclear cells (PBMCs) from C57BL/6 (B6), B6. Cd2−/−, Balb/c and Balb/c. Peripheral blood mononuclear cells (PBMCs) from C57BL/6 (B6), B6. Cd2−/−, Balb/c, and Balb/c. Cd2−/− mice were analyzed for surface expression of (A) CD2 and TCRβ and (B) H2kb and H2kd by flow cytometry. Representative density plots from at least 4 mice per genotype are shown. [file Image_1.JPEG]

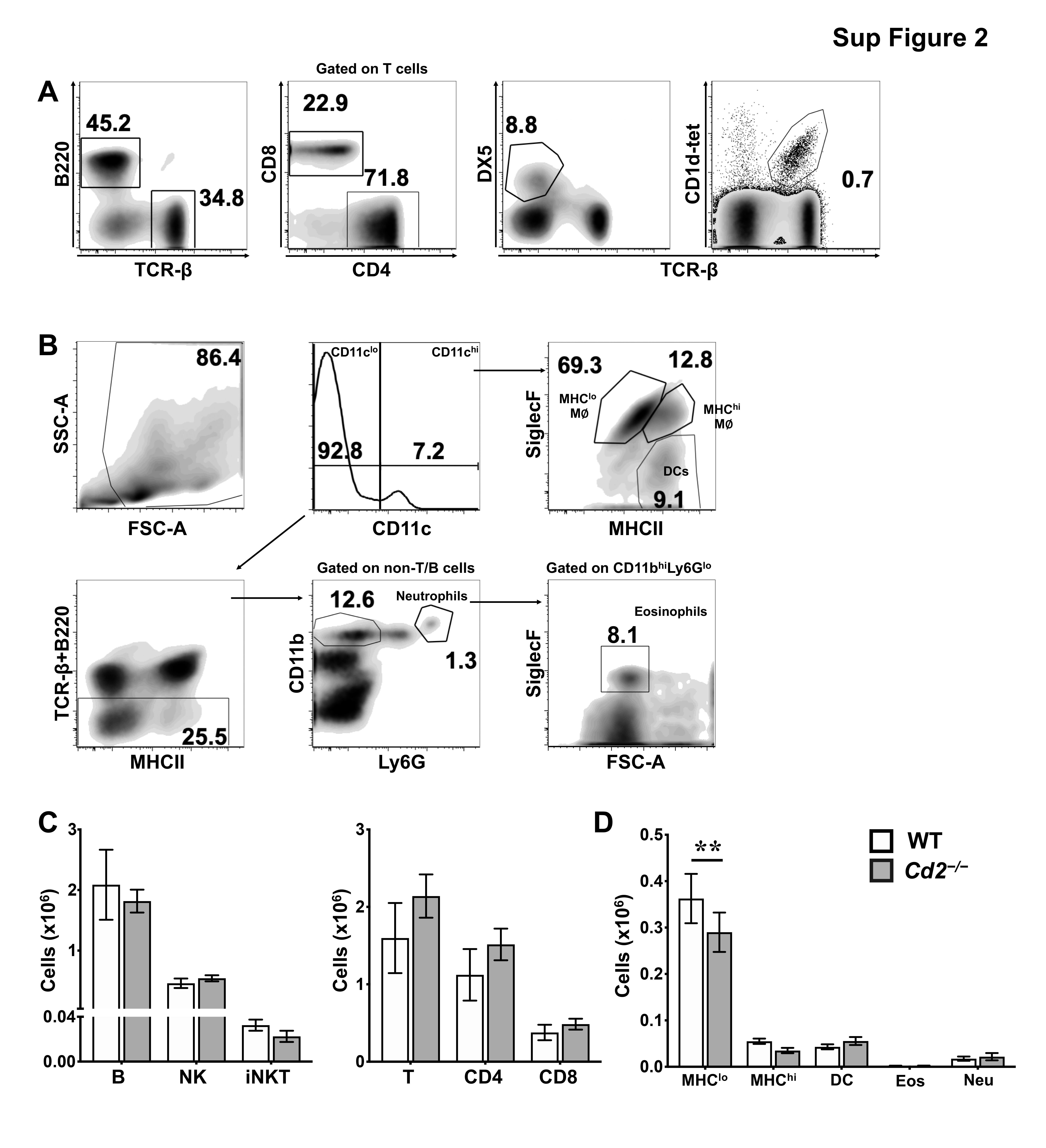

Supplement: Supplemental Figure 2 — Cd2−/− mice have normal immune cell distribution. (A,B) Representative flow cytometry analysis showing various lymphocyte (B, NK, iNKT, T, CD4 T, CD8 T cell) populations (A), and gating strategy for innate cells (B) in lungs of naïve WT mice, as indicated. (C,D) Absolute numbers of lymphocytes (C) and innate cells (D) in lungs of naïve WT and Cd2−/− mice. Data shown is representative of 4 mice for each genotype. Statistical significance was determined by Student's t test with Welch's correction. *p ≤ 0.05, **p ≤ 0.01. [file Image_2.JPEG]

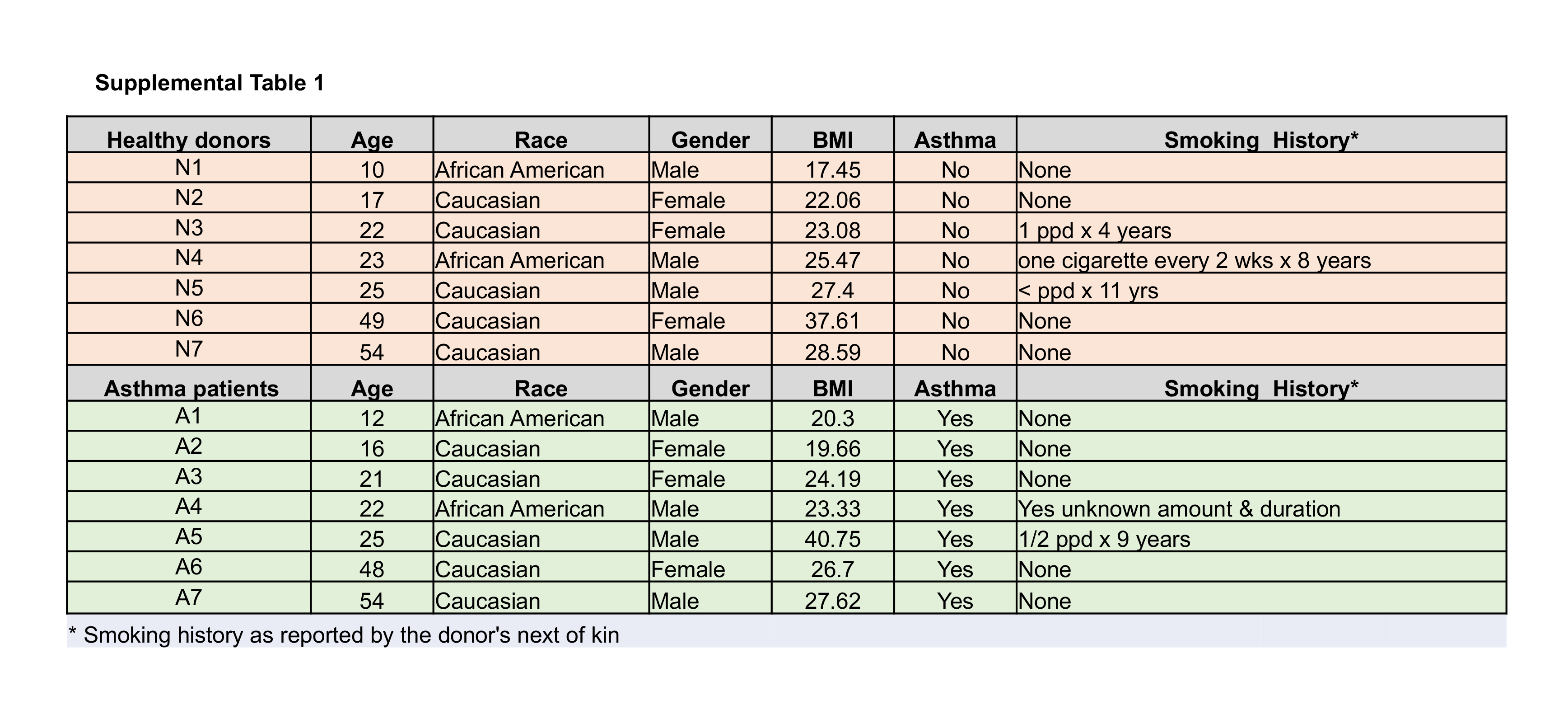

Supplement: Supplemental Table 1 — Asthma patient and normal donor information. [file Image_3.JPEG]

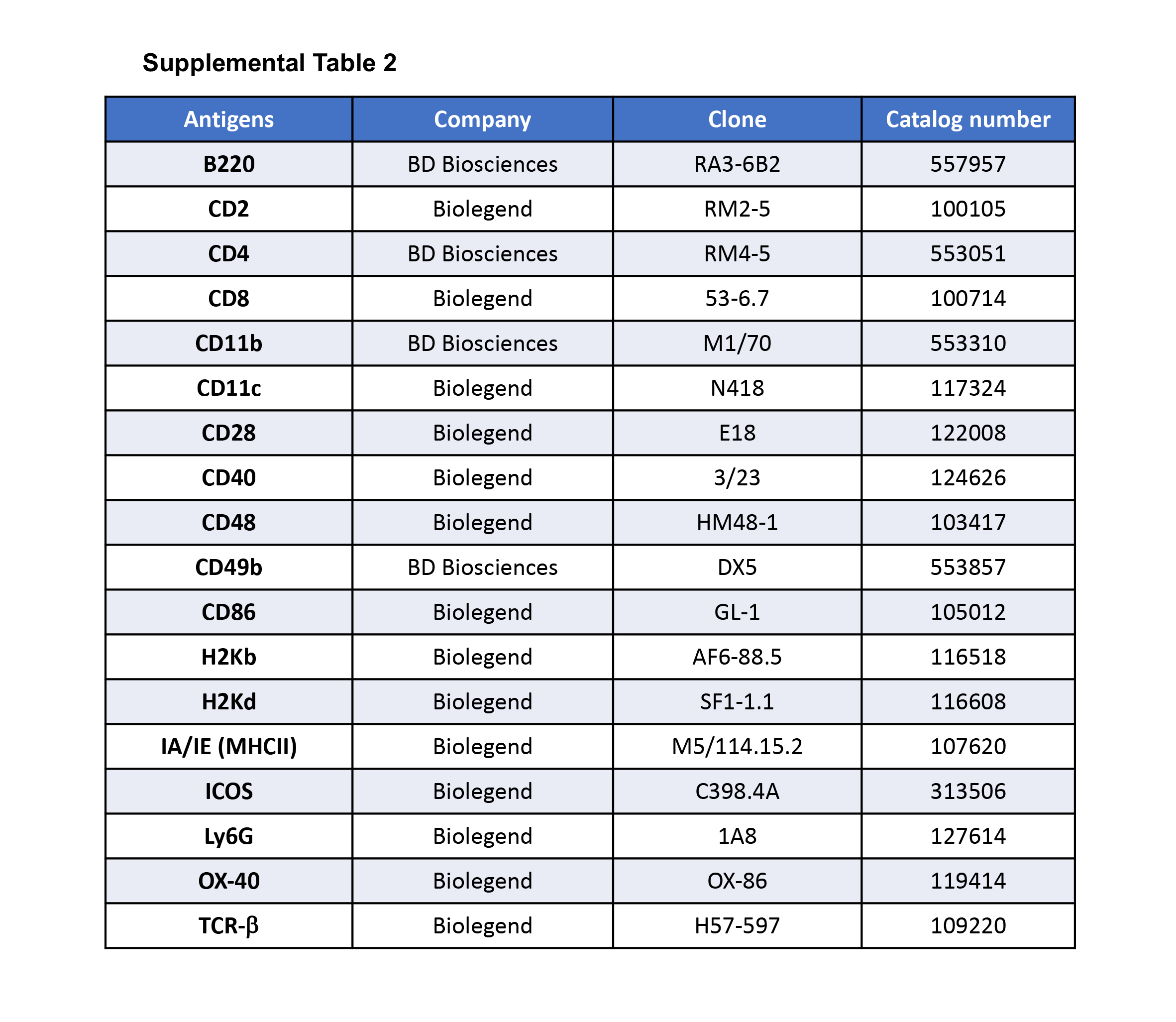

Supplement: Supplemental Table 2 — Antibodies used for flow cytometry. [file Image_4.JPEG]
